# The Association of American Medical Editors

**Published:** 1879-06

**Authors:** 


### The Association of American Medical Editors.

In the Senate Chamber of the Capitol at Augusta, Georgia, May 5, 1879, the Association of American Medical Editors held its eleventh annual meeting on the evening before the session of the American Medical Association, fifteen journals being represented.

The retiring president, Dr, Wm. Brodie, delivered an address on Medical Journalism, in which he strongly condemned the practice of allowing patent medicine to be advertised in reputable medical journals, and denounced it as contrary to the spirit of the Code of Ethics. He concluded by offering a series of resolutions expressing this sentiment. On motion of Dr. Dunster, the resolutions were adopted, and ordered to be presented to the American Medical Association. Dr Parvin urged the publication of details of cases by country physicians, as a means of increasing our knowledge of clinical medicine. He announced the death of two medical editors, Dr. Isaac Hayes and Dr. Waddell, and, upon his motion, a committee was appointed to present a proper memorial upon this subject. The Committee on Nominations reported the following ticket for officers, which was unanimously elected, the secretary casting the ballot for the Association:

President, Dr. Thomas S. Powell, *Southern Medical Record*, Atlanta, Ga.

Vice-Presidents, Dr. Frank Woodbury (Philadelphia), *Boston Medical and Surgical Journal.*

Secretary, Dr. Frank Davis, *Chicago Medical Journal.*

Place of meeting in 1880, New York city. The editors of the *Southern Medical Record* gave a dinner to the Association on the 7th of May, at the residence of Dr. Powell, which greatly enhanced the pleasure of the meeting, and extended the opportunities for acquaintance and social intercourse, the visitors carrying away with them a warm appreciation of Georgia hospitality.

———:o:———

